# Development of an Optical Method to Monitor Nitrification in Drinking Water

**DOI:** 10.3390/s21227525

**Published:** 2021-11-12

**Authors:** Sharif Hossain, David Cook, Christopher W. K. Chow, Guna A. Hewa

**Affiliations:** 1 Scarce Resources and Circular Economy (ScaRCE), UniSA STEM, University of South Australia, Adelaide 5095, Australia; christopher.chow@unisa.edu.au (C.W.K.C.); guna.hewa@unisa.edu.au (G.A.H.); 2 South Australian Water Corporation, Adelaide 5000, Australia; david.cook@sawater.com.au; 3 Future Industries Institute, University of South Australia, Adelaide 5095, Australia

**Keywords:** optical nitrification monitoring, UV-Vis absorbance spectra, spectrophotometric analysis of nitrate and nitrite, spectral compensation, drinking water nitrification monitoring

## Abstract

Nitrification is a common issue observed in chloraminated drinking water distribution systems, resulting in the undesirable loss of monochloramine (NH_2_Cl) residual. The decay of monochloramine releases ammonia (NH_3_), which is converted to nitrite (NO_2_^−^) and nitrate (NO_3_^−^) through a biological oxidation process. During the course of monochloramine decay and the production of nitrite and nitrate, the spectral fingerprint is observed to change within the wavelength region sensitive to these species. In addition, chloraminated drinking water will contain natural organic matter (NOM), which also has a spectral fingerprint. To assess the nitrification status, the combined nitrate and nitrite absorbance fingerprint was isolated from the total spectra. A novel method is proposed here to isolate their spectra and estimate their combined concentration. The spectral fingerprint of pure monochloramine solution at different concentrations indicated that the absorbance difference between two concentrations at a specific wavelength can be related to other wavelengths by a linear function. It is assumed that the absorbance reduction in drinking water spectra due to monochloramine decay will follow a similar pattern as in ultrapure water. Based on this criteria, combined nitrate and nitrite spectra were isolated from the total spectrum. A machine learning model was developed using the support vector regression (SVR) algorithm to relate the spectral features of pure nitrate and nitrite with their concentrations. The model was used to predict the combined nitrate and nitrite concentration for a number of test samples. Out of these samples, the nitrified sample showed an increasing trend of combined nitrate and nitrite productions. The predicted values were matched with the observed concentrations, and the level of precision by the method was ± 0.01 mg-N L^−1^. This method can be implemented in chloraminated distribution systems to monitor and manage nitrification.

## 1. Introduction

Nitrate (NO_3_^−^) and nitrite (NO_2_^−^) ions are the most common forms of inorganic nitrogen found in environmental samples [1,2]. The abundance and distribution of these species in surface water are often characterized by hydrological regime, seasonal changes, spatial and temporal variability, and the mode and nature of the nutrient sources [1]. They are regulated pollutants, and drinking water treatment may be required to reduce their level within the regulated limits. Nitrification is a common issue observed in drinking water disinfected by monochloramine (NH_2_Cl) [3,4]. This is a microbiological process where free ammonia (NH_3_), released through monochloramine decomposition, is converted to nitrite and nitrate by nitrifying bacteria, resulting in elevated concentrations of these species in water [3,5]. Drinking water containing high levels of nitrate and nitrite may pose threats to public health. The World Health Organization suggests that the maximum contaminant level (MCL) of nitrate and nitrite in drinking water should not exceed 11.3 mg L^−1^ as NO_3_-N, and 0.9 mg L^−1^ as NO_2_-N [6,7]. Fast and rapid detection of nitrate and nitrite is required to minimize the risk of nitrification, and to ensure the regulatory compliance [8].

Analytical methods used to determine nitrate and/or nitrite include colorimetry, potentiometry, spectrophotometry, spectrofluorimetry, chromatography, etc. [9,10]. Over many years, optical techniques have been successfully used to measure a range of water quality parameters, including dissolved organic carbon (DOC), nitrate, nitrite, and monochloramine, with an excellent limit of detection [11,12,13,14]. Singh et al. [2] reviewed various spectroscopic methods and concluded that UV-Vis spectrophotometry is widely accepted because of its simplicity, feasibility, and versatility. Evidence described in the literature suggests that inorganic nitrate and nitrite ions dissolved in water strongly absorb UV light, with a high correlation between concentration and absorption spectra [1,8,13,15,16]. The inherent absorption properties of nitrate and nitrite will make it possible to detect them using the spectrophotometric method without using any reagents [13]. For nitrite, the absorption peak appears around 213 nm, whereas for nitrate, different wavelengths for absorption peak are reported that vary by concentration [16,17]. The absorption peak of nitrate gradually shifts to the right as the concentration increases. Karlsson et al. [8] reported that nitrate has an absorption peak at 205 nm wavelength. A similar study reported that nitrate has a primary and secondary peak at 202 nm and 302 nm, respectively. In contrast, for nitrite, the primary and secondary peaks appeared at 210 nm and 355 nm, respectively [11]. However, at a low level of nitrate and nitrite usually produced in typical chloraminated drinking water during nitrification, the secondary peak is unsuitable to detect and measure because of the inadequate response at that wavelength. Measuring them using the primary peak is also often disturbed by interference by other organic and inorganic substances that absorb UV light within the primary peak region. As both nitrate and nitrite absorb UV light in the same wavelength range, the global NO_x_^−^ concentration (combined NO_2_^−^ + NO_3_^−^) is sometimes preferred to avoid the difficulties associated with discriminating between nitrate and nitrite [13].

Several studies have reported the application of different data analytic techniques for the determination of nitrate and nitrite using single or multi-wavelength absorption spectra [1,8,11,13,15,17,18,19,20,21,22]. For instance, Karlsson et al. [8] used multivariate data analysis to relate spectra and concentrations of municipal wastewater. A different approach was proposed by Jiao et al. [15] who used the secondary absorption peak to link spectra and nitrite content in environmental samples. Using multiple linear regression, they assessed the effect of pH on regression performance, where the best modelling performance was achieved at a pH of 5 or greater because the component of aqueous solution of nitrite depends on the pH value. When the pH is 5 or above, the solutions contain more than 99% of nitrite, whereas if the pH is 1.1 or below, the solution contains more than 99% of nitrous acid (HONO) [23]. The second derivative of absorbance (SDA) is also widely used to resolve the overlapping peaks, and to enhance the signal [1,24]. Based on the SDA method, Ferree and Shannon [19] measured the nitrate content in wastewater samples, whereas Causse et al. [1] assessed the SDA at 226 nm to determine the same in raw waters. Similarly, Pons et al. [22] assessed a range of river water samples and found good linear correlation between the maxima of the SDA and the corresponding nitrate concentrations. In typical drinking water, natural organic matter (NOM) absorbs a significant portion of UV light, causing major interference towards the measurement of nitrate and nitrite. Hence, using more than one wavelength, where both nitrate and nitrite do not absorb, can improve the measurement accuracy. Edwards et al. [25] proposed absorbance measurements at 205 nm and 300 nm to determine nitrate content in the presence of NOM. Huebsch et al. [17] evaluated the double and multi-wavelength spectrophotometric measurements using partial least square regression (PLSR) for monitoring nitrate in groundwater samples. These studies suggested that UV-Vis spectrophotometry coupled with various data analytic techniques can be used as a powerful tool to measure the nitrate, nitrite, or combined NO_x_^−^ concentrations in water from various sources, including drinking water, river water, waste water, etc.

When a chloraminated sample undergoes nitrification, significant changes in various water quality parameters are evident [3,4]. This includes a decrease in monochloramine concentration, an increase in NO_x_^−^ production, and an initial increase of ammonia concentration followed by a decrease. The water quality change suggests that the method needs to be sensitive, such that changes in NO_x_^−^ and monochloramine as a result of nitrification can be detected. Theoretically, the NOM concentration, measured as dissolved organic carbon (DOC), should also change. However, the change of DOC concentration over time is relatively slow and small in magnitude, whereas ammonia has little or no UV absorbances under the typical range of concentration found in drinking water. Hence, the contribution of NOM and ammonia to change the spectra can be ignored. When no monochloramine residual is left in water, the NO_x_^−^ production between two timestamps, t = 0 and t = t, is related to the NO_x_^−^ spectra, which can be simply obtained by subtracting the spectra at t = 0 from the spectra at t = t. However, if there is monochloramine residual left in water, the spectra difference between two timestamps does not represent the whole NO_x_^−^ spectra, as monochloramine has absorbance within the range absorbed by NO_x_^−^ species. In this case, an additional spectral compensation is required to modify the spectra interfered by monochloramine absorbances. This study proposes such a spectral compensation to account the monochloramine absorbance, based on the behavior of pure monochloramine spectra. Hence, the objectives of this study are: (i) to isolate the NO_x_^−^ spectra from the total spectra; and (ii) to determine the NO_x_^−^ concentrations using standard data analytic techniques. This method works on determining the relative NO_x_^−^ production between two timestamps, hence, it is suitable to use when there is no additional information of lab NO_x_^−^ is available. This is a novel method, as no previous studies offer a similar approach to what is proposed in this paper. Hence, this paper provides a significant new knowledge contribution to manage nitrification in drinking water systems.

## 2. Materials and Methods

### 2.1. Analytical Methods

Turbidity was measured using a Hach TU5200 turbidimeter (Hach, Loveland, CO, USA), and the measurement was obtained as nephelometric turbidity units (NTU). pH and temperature were measured using a portable pH meter (pH320, WTW, Weilheim, Germany). Free chlorine, monochloramine, and dichloramine were determined using the *N*,*N*-diethyl-p-phenylenediamine (DPD)-ferrous ammonium sulfate (FAS) titrimetric procedure (APHA 4500-CI-F) [26]. UV-Vis absorbance was measured using an Evolution 300 spectrophotometer (Thermo Scientific, Waltham, MA, USA) through a 10 cm quartz cell. This is a double beam spectrophotometer capable of acquiring spectra from 200 nm to 800 nm. The data resolution was set to 0.5 nm. Samples were prepared for spectral analysis by filtering them through 0.45 μm filter paper in order to remove the particle absorbances. The DOC concentration was measured using a Sievers M5310C Total Organic Carbon (TOC) analyzer fitted with an auto sampler (GE Analytical Instruments, Boulder, CO, USA). The dissolved species were defined by those that could pass through the 0.45 μm filter paper. Ammonia concentration was measured using the ion-selective ammonia electrode method (APHA 4500-NH_3_-D) [26]. Nitrate and nitrite were determined by the automated flow colorimetry method (APHA 4500-NO_3_-I) [26].

### 2.2. Research Methodology

**Figure 1:**
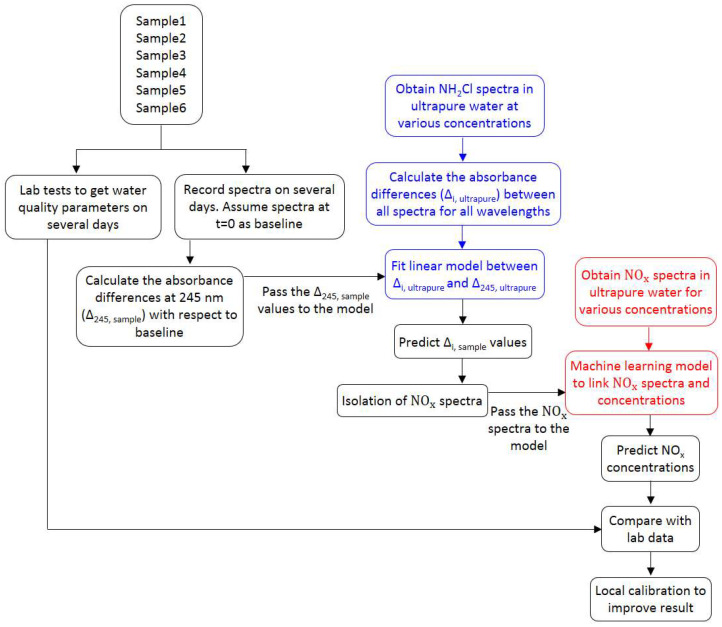
Framework of the study. The blue and red colors represent individual experiments related to monochloramine and NO_x_^−^,respectively.

The systematic procedure adopted in the research is presented in Figure 1.

**Figure 2:**
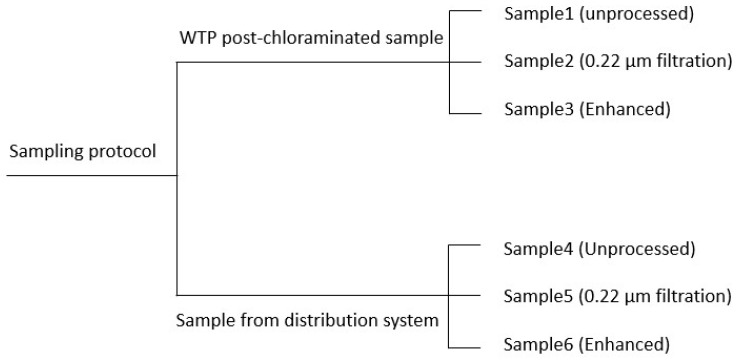
Sample preparation using the grab water collected from WTP and the distribution system.

The study tested water samples from the Tailem Bend to Keith drinking water distribution system located in South Australia. Grab samples were collected from two different locations. The first location was the Tailem Bend water treatment plant (TB-WTP) (post-chloraminated water), and the second was a location in the distribution system that had experienced rapid chloramine decay and nitrification several times in the past years. Using the collected waters, six different samples were prepared. Sample1 and Sample4 were made up by the original water from these locations, whereas Sample2 and Sample5 were obtained by filtering the original waters through 0.22 μm filter paper. The purpose of the filtering was to remove a portion of microbes/nitrifying bacteria, in order to limit the nitrate and nitrite production. Sample3 and Sample6 were prepared to enhance the microbiological activity by adding the previously obtained 0.22 μm filter retained materials and 0.8 mg L^−1^ of NH_3_-N. Bacteria was retrieved from the filter by simply rinsing the filter paper with the sample water. The test strategy covers wide variations in water characteristics, allowing different levels of nitrate and nitrite accumulation during the course of the study. The whole sampling strategy is presented in Figure 2.

**Figure 3:**
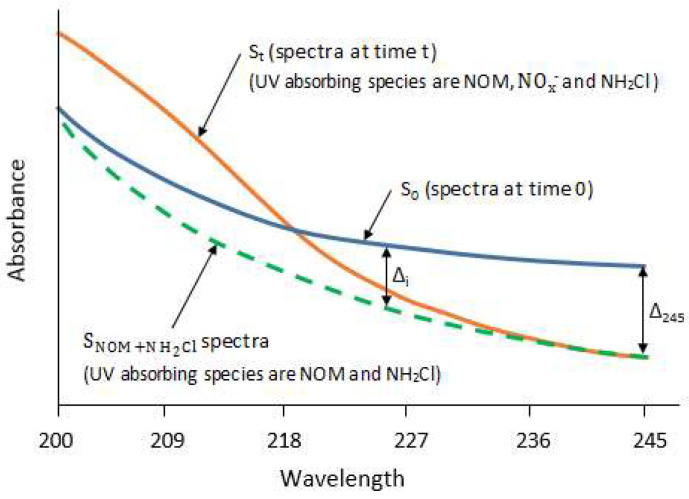
Procedure of estimating the spectra at time t subjected to monochloramine decay only.

The samples were kept in a dark place under normal room temperature. The spectra of these samples were recorded for several weeks. At the same time, various water quality parameters, such as monochloramine, dichloramine, free ammonia, DOC, nitrate, nitrite, etc., were also analyzed on a regular basis. For each sample, the first spectra (t = 0) were considered as baseline, and are represented by S_0_. It has been reported that monochloramine has a UV absorbance peak at 245 nm [14,27]. Therefore, due to monochloramine decay, the spectra will shift downward from the baseline, with maximum offset at the peak. For modelling purposes, the criteria to find a suitable offset was defined by (i) maximum offset and (ii) minimum nitrate and nitrite absorbances. The maximum offset will minimize the error when using it as a model predictor, whereas minimum nitrate and nitrite absorbances will ensure the calculation of the true variation of spectra due to monochloramine decay only. Data analysis suggests that an offset at 245 nm satisfy both criteria. In parallel, the monochloramine decay will produce ammonia, which may further undergo microbiological reactions to produce nitrite and nitrate. Both of the nitrogen species have high UV absorbance between 200 nm and 230 nm. Consequently, the spectra will shift upward from the baseline. Hence, the recorded spectra at any time t, referred to as S_t_, is characterized by the absorbance contribution by NOM, monochloramine, and NO_x_^−^. The term NO_x_^−^ spectra refers to the combined nitrate and nitrite spectra. To isolate the NO_x_^−^ spectra from S_t_, it is required to approximate the spectra at time t due to monochloramine decay only, referred to as $S_{\mathrm{NOM}+{\mathrm{NH}}_{2}\mathrm{Cl}}$. This was done by investigating the monochloramine concentration absorbance relationship of all wavelengths to 245 nm in ultrapure water. Figure 3 shows the procedure where the value of ∆_245_ is known, whereas its values for other wavelengths (∆_i_) need to be estimated to calculate the spectra at time t resulting from monochloramine decay only.

To assess the spectral characteristics of pure monochloramine, a standard solution at a concentration of 10 mg L^−1^ was prepared using ultrapure water (Millipore, Molsheim, France). Through successive dilution, a range of monochloramine solutions of concentration, ranging from 0.2 mg L^−1^ to 5 mg L^−1^, were prepared, and their corresponding spectra were recorded. Linear models were used to relate the spectra differences at 245 nm (∆_245,ultrapure_) with the spectra differences at other wavelengths (∆_i,ultrapure_). The relationship between ∆_245,ultrapure_ and ∆_i,ultrapure_ was investigated at three different pH of 7, 8, 9, and was found to not change significantly within that pH range. The regression function can be expressed by the following equation:

(1) $$\Delta_{\;i,\;\mathrm{ultrapure}}=\mathrm{Linear}\;f\;\left(\Delta_{\;245,\;\mathrm{ultrapure}}\right)\;\mathrm{for}\;i=200\;\mathrm{nm}\;\mathrm{to}\;244.5\;\mathrm{nm}$$

where $\Delta_{\;245,\;\mathrm{ultrapure}}$ is the set of monochloramine spectra differences between various concentrations at 245 nm in Milli-Q water, and $\Delta_{\;i,\;\mathrm{ultrapure}}$ is the set of corresponding spectra differences at the i^th^ wavelength. The value of i ranges from 200 nm to 244.5 nm, as the spectra recording resolution was set to 0.5 nm. 

It was assumed that chloraminated drinking water will follow the same ∆_i_ pattern as in Milli-Q water. Therefore, these models were used to predict the $\Delta_{i,\;\mathrm{sample}}$ values with $\Delta_{245,\;\mathrm{sample}}$ being predictors. These predicted ∆_i_ values were subtracted from the baseline to approximate the spectra caused by the monochloramine decay effect only ($S_{\mathrm{NOM}+{\mathrm{NH}}_{2}\mathrm{Cl}}$). This estimated $S_{\mathrm{NOM}+{\mathrm{NH}}_{2}\mathrm{Cl}}$ spectra do not include the absorbances by the NO_x_^−^ component. It should be noted that NOM may have some minor changes in concentrations during the time t, and can affect the $S_{\mathrm{NOM}+{\mathrm{NH}}_{2}\mathrm{Cl}}$ spectra. However, DOC analysis suggests that NOM concentration is relatively stable compared to monochloramine, and the subsequent effect can be ignored. Further, subtracting the $S_{\mathrm{NOM}+{\mathrm{NH}}_{2}\mathrm{Cl}}$ spectra from the sample spectra at time t, S_t_ will give the NO_x_^−^ spectra.

To extract the combined nitrate and nitrite concentrations from the NO_x_^−^ spectra, standard light absorbances by these species in ultrapure water need to be assessed. For this purpose, standard solutions were prepared. A nitrate standard solution at a concentration of 100 mg L^−1^ as NO_3_^−^-N was prepared by dissolving 0.7218 gm dry potassium nitrate (KNO_3_) to ultrapure water, and diluted to 1000 mL. Similarly, a nitrite standard solution (100 mg L^−1^ as NO_2_^−^-N) was prepared by dissolving 0.15 gm of sodium nitrite (NaNO_2_) to Milli-Q water, and diluted to 1000 mL. From these standard solutions, different concentrations of nitrate and nitrite solutions were prepared using appropriate dilution factors. 

The NO_x_^−^ spectra and the known nitrate and nitrite concentrations data were used to develop a machine learning model using the support vector regression (SVR) algorithm. Absorbances at various wavelengths were considered as predictor variables, whereas the corresponding concentrations were assumed to be the response variables. The theory and application of the SVR algorithm is available in many pieces of literature [14,28,29]. In brief, the SVR algorithm is an extension of the support vector classification (SVC), where a hyperplane is defined to classify two classes of data. From both sides of the hyperplane, an epsilon(ε) range is assigned where the regression function is considered to be insensitive. The support vectors are the data points closer to the hyperplane. For a multi-class classification problem, the SVC algorithm uses a series of binary classifiers to perform the classification. If the hyperplane cannot separate the data linearly, SVC uses a kernel trick to transform the data into higher dimensions where a linear separation exists. The four major types of kernel functions commonly used in the SVR algorithm are: (i) linear; (ii) polynomial, (iii) radial basis function (RBF); and (iv) sigmoid. The accuracy of the SVR model can be improved by tuning the model parameters and choosing the appropriate kernel function. The SVR model training performance was validated using a 10-fold cross-validation. Data were scaled in between +1 and -1 to ensure the SVR convergence. The goodness-of-fit in model training and cross-validation were assessed using the coefficient of determination (R^2^) and root mean square error (RMSE). They were calculated by the following formulae:

(2) $$\mathrm{Coefficient}\;\mathrm{of}\;\mathrm{determination}\;\left(R^{2}\right)={\left[\frac{\sum_{i=1}^{n}\left(O_{i}-\bar{O}\right)\left(P_{i}-\bar{P}\right)}{\sqrt{\sum_{i=1}^{n}{\left(O_{i}-\bar{O}\right)}^{2}}\sqrt{\sum_{i=1}^{n}{\left(P_{i}-\bar{P}\right)}^{2}}}\right]}^{2}$$

(3) $$\mathrm{Root}\;\mathrm{mean}\;\mathrm{square}\;\mathrm{error}\;\left(\mathrm{RMSE}\right)=\sqrt{\frac{\sum_{i=1}^{n}{\left(P_{i}-O_{i}\right)}^{2}}{n}}$$

where $O_{i}$ and $P_{i}$ are the observed and predicted values of the ${\mathrm{NO}}_{x}$^−^ concentrations, respectively, *n* is the number of data points, and $\bar{O}$ and $\bar{P}$ are the observed and predicted mean values. The value of R^2^ lies between 0 to 1, where 1 indicates that the model can describe 100% of the variation in the data. In contrast, 0 means that model prediction is not any better than the mean of the data [30,31]. The value of RMSE indicates how widely the residuals (difference between the modelled and the observed data) disperse. A relatively low RMSE value indicates a better fit [30,31].

Finally, the developed SVR model was used to predict the relative NO_x_^−^ concentrations in several test samples using their isolated NO_x_^−^ spectra. A local calibration was done to improve the NO_x_^−^ estimation accuracy. In the local calibration, a linear/non-linear relationship is developed between the observed lab data and the model predicted values.

## 3. Results

The average water quality parameters at the TB-WTP and the selected location of the distribution system (Meningie) are presented in Table 1. These values were obtained through grab sample analysis at the laboratory. 

As shown in Table 1, most water quality parameters, including pH, turbidity, DOC, free chlorine and dichloramine concentrations, are similar at both locations. However, monochloramine, free ammonia and NO_x_^−^ concentrations vary significantly. For instance, the typical monochloramine concentration at the WTP is 4.10 ± 0.30 mg L^−1^, whereas at Meningie, the concentration drops to 1.60 ± 1.0 mg L^−1^. A significant amount of monochloramine residual is lost along the way to Meningie. At the same time, free ammonia and NO_x_^−^ concentrations at Meningie also increased. Decreased monochloramine residual and increased free ammonia and NO_x_^−^ concentrations show the incidences of nitrification at Meningie several times in the past years.

**Figure 4:**
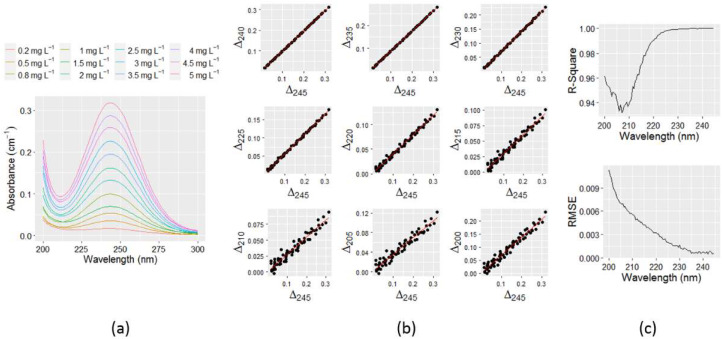
(**a**) Pure monochloramine spectra in Milli-Q water with different concentrations, ranging from 0.2 mg L^−1^ to 5.0 mg L^−1^; (**b**) plot of absorbance difference at various wavelengths against the same at 245 nm; and (**c**) R^2^ and RMSE values in linear model fit between $\Delta_{245,\;\mathrm{ltrapure}}$ and $\Delta_{i,\;\mathrm{ultrapure}}$.

The typical monochloramine spectra presented in Figure 4a shows the UV absorbances in Milli-Q water of up to 300 nm for concentrations ranging from 0.2 mg L^−1^ to 5.0 mg L^−1^. Water utilities usually practice chloramination within that range. The remaining region of the spectra is relatively flat and not shown. The peak absorption appeared at 245 nm wavelength, which aligns with many previous studies [14,27]. For all concentrations tested, the spectra were very symmetric, with a high correlation between concentrations and absorbances at 245 nm wavelength observed. For the formation of monochloramine in Milli-Q water, the pH was initially adjusted to 9 to minimize the formation of dichloramine, as a relatively high pH can potentially reduce the dichloramine formation [32,33]. Consequently, the interference by the dichloramine spectra was the minimum. A relatively high pH value also ensured no significant monochloramine autodecomposition occurred during the course of the experiment [32]. After chloramination, the measured dichloramine concentration was less than 0.1 mg L^−1^. Therefore, the spectra obtained were assumed to be the pure monochloramine spectra without any significant interference by dichloramine. As the pH of chloraminated drinking water samples can vary throughout the distribution system, the pure monochloramine spectra were also investigated for two other pH of 8 and 7. Using the spectral data at various pH combinations allows a reliable estimate to be determined for a range of values at an unknown pH. However, no significant differences were found in absorbance spectra between pH values of 7 and 9.

As can be seen in Figure 4a, the spectra are very symmetric about the concentrations, hence, the absorbance differences (∆_I,ultrapure_) for two different monochloramine concentrations at various wavelengths can be related to each other by some functions. Newly composed R programming codes were used to calculate ∆_i,ultrapure_ for all possible combinations of concentrations at each wavelength from 200 nm to 245 nm. The wavelength 245 nm was chosen because monochloramine peak absorption wavelength approximately appears at 245 nm, hence, maximum absorbance difference (∆_max_) is expected at that wavelength (∆_max,ultrapure_ = ∆_245,ultrapure_). Therefore, expressing ∆_i,ultrapure_ as a function of ∆_245,ultrapure_ will encounter a relatively lower error in regression. Moreover, typical nitrate and nitrite spectra do not have significant UV absorbances beyond 245 nm. The relationship between ∆_i,ultrapure_ and ∆_245,ultrapure_ can be determined by plotting these data and fitting the standard trend line. Figure 4b shows the plot of ∆_i,ultrapure_ against ∆_245,ultrapure_ at many intermediate wavelengths from 240 nm to 200 nm with a 5 nm increment. The trend lines in these plots indicate that there is a linear association between ∆_i,ultrapure_ and ∆_245,ultrapure_. Figure 4c shows the R^2^ and RMSE values in the linear model fit, where it is evident that the value of R^2^ gradually improved, and RMSE decreased towards 245 nm. The lowest R^2^ obtained in the linear model fit was greater than 0.93, which indicates that the variable ∆_245,ultrapure_ can adequately predict the ∆_i,ultrapure_ with a good level of accuracy. The full range of R^2^ and RMSE values between 200 nm and 244.5 nm is presented in Table A1 in Appendix A.

**Figure 5:**
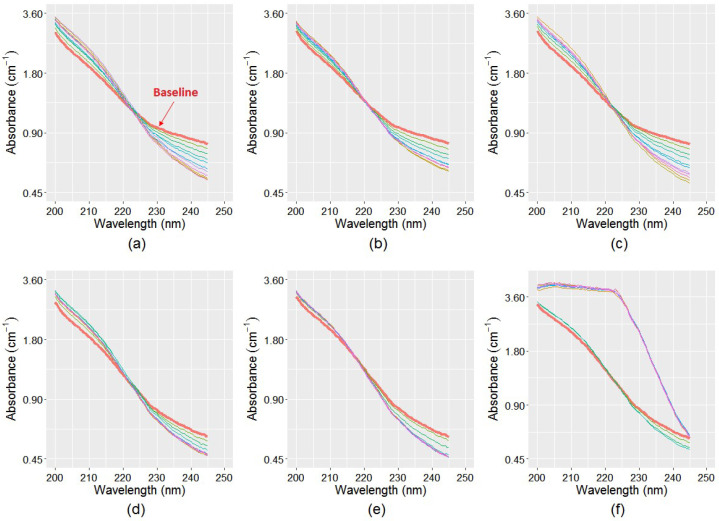
Recorded spectra of the six samples for a period of eight weeks: (**a**) TB-WTP unprocessed sample; (**b**) TB-WTP 0.22 μm filtered sample; (**c**) TB-WTP enhanced sample; (**d**) Meningie unprocessed sample; (**e**) Meningie 0.22 μm filtered sample; and (**f**) Meningie enhanced sample. Nitrification was observed in Sample6 (Figure 5f). Due to NO_x_^−^ production, the subsequent spectra in all samples cross the baseline in between 200 nm and 225 nm.

The recorded spectra for samples1–6 during a period of eight weeks is shown in Figure 5. For each sample, the initial spectra (t = 0) were considered as baseline. As can be seen in Figure 5, the absorbances at 245 nm decreased in all samples over time due to monochloramine decay, whereas it increased between 200 nm and 225 nm due to nitrate and nitrite productions. All sample spectra intersect their baseline spectra at approximately 220 nm, which also indicated that, except monochloramine, there are absorbances by other species involved in the spectra, which was nitrate and nitrite. Due to the monochloramine decay effect, the whole spectra will shift proportionally downward throughout 200 nm to 245 nm, and will not cross the baseline. The highest variability in spectral configuration was found in the enhanced samples (Sample3 and Sample6). The starting monochloramine concentration of these samples were 4.3 mg L^−1^ at the TB-WTP site (Samples1–3) and 1.3 mg L^−1^ at the Meningie site (Samples4–6), respectively. Nitrification was observed in Sample6, where a significant increase in UV absorbance was evident. It is possible that Sample3 did not experience nitrification because its initial monochloramine concentration was relatively higher compared to that of Sample6. Thus, the microbial inactivation rate by monochloramine was also much higher compared to their growth rate. On the other hand, Sample4 and Sample5 have near similar spectral changes around the 245 nm region, but different level of changes in between 200 nm and 225 nm, indicating different levels of NO_x_^−^ productions in these samples. This suggests that NO_x_^−^ production is related to a number of factors, including initially available ammonia, the production of additional ammonia through monochloramine decomposition, and the rate of ammonia conversion to NO_x_^−^.

**Figure 6:**
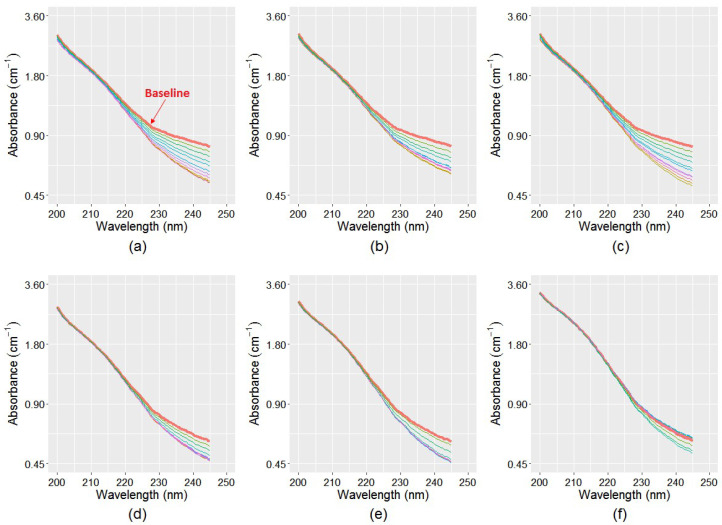
Estimated spectra without the influence of NO_x_^−^ absorbances for the six samples for a period of eight weeks: (**a**) TB-WTP unprocessed sample; (**b**) TB-WTP 0.22 μm filtered sample; (**c**) TB-WTP enhanced sample; (**d**) Meningie unprocessed sample; (**e**) Meningie 0.22 μm filtered sample; and (**f**) Meningie enhanced sample. Spectral change is assumed to be caused by monochloramine decay only. Compared to Figure 5, the subsequent spectra in all samples in Figure 6 do not cross the baseline, as no NO_x_^−^ absorbances are considered.

With regard to the baseline, the absorbance difference at 245 nm wavelength, ∆_245, sample_, was calculated for each sample, which was then used to predict the ∆_i, sample_ values using the linear functions between ∆_245, ultrapure_ and ∆_i, ultrapure_. This produces a dataset of n − 1 sets of ∆_200–245_ values for n number of spectra at each sample. These estimated ∆_i, sample_ values were subtracted from their baseline values to estimate the spectra change caused by the monochloramine decay only. Figure 6 shows the estimated spectra for the six samples, where the topmost spectra are the baseline. It is evident in the figure that spectra sets in Samples1–3 (Figure 6a–c) have much downward shift compared to that observed in Samples3–6 (Figure 6d–f). This is because initial monochloramine concentration of Samples1–3 was much higher compared to Samples3–6, hence, more absorbance reduction occurred in these samples because of monochloramine decay during the study period. 

**Figure 7:**
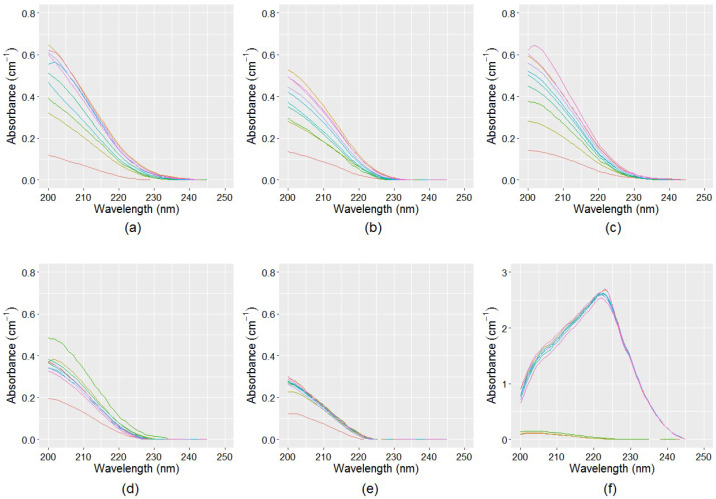
Isolated NO_x_^−^ spectra for a period of eight weeks: (**a**) TB-WTP unprocessed sample (sample1); (**b**) TB-WTP 0.22 μm filtered sample (sample2); (**c**) TB-WTP enhanced sample (sample3); (**d**) Meningie unprocessed sample (sample4); (**e**) Meningie 0.22 μm filtered sample (sample5); and (**f**) Meningie enhanced sample (sample6).

For all samples, the spectra in Figure 6 were subtracted from the spectra in Figure 5 to obtain the NO_x_^−^ spectra that includes both nitrate and nitrite absorbances. The isolated NO_x_^−^ spectra presented in Figure 7 show that the enhanced samples (Sample3 and Sample6) have the maximum UV absorbances, suggesting that the production of nitrate and nitrite are the maximum in these samples. This is because the ammonia concentration was increased in these samples to encourage microbiological activity, resulting in an increased conversion of ammonia to nitrate and nitrite. Further, some spectra in Sample6 showed an increase in absorbance compared to other samples by several orders of magnitude, suggesting that severe nitrification occurred. In contrast, the NO_x_^−^ spectra in filtered samples (Sample2 and Sample5) have the lowest absorbance response, hence, nitrification was absent in these samples because microbiological activity was reduced by filtering these samples. Compared to filtered and enhanced samples, the unprocessed samples (Sample1 and Sample4) have an intermediate level of NO_x_^−^ production. The isolated NO_x_^−^ spectra closely resemble the typical NO_x_^−^ spectra in Milli-Q water.

**Figure 8:**
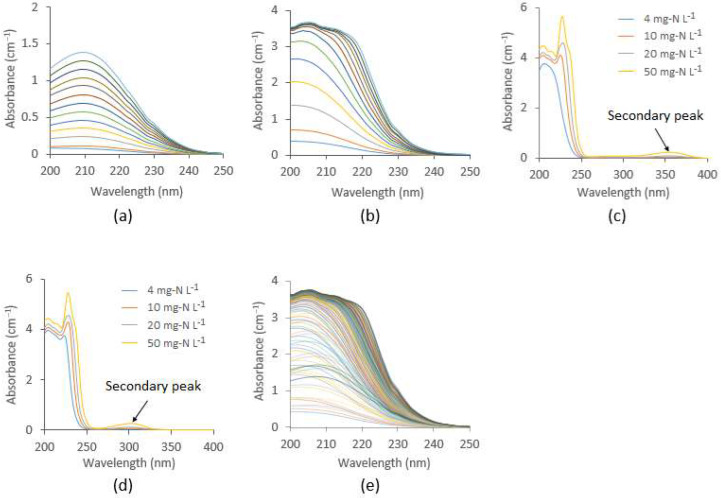
(**a**) Pure nitrite spectra around the primary peak region within concentration range of 0.001 mg-N L^−1^ to 1.2 mg-N L^−1^; (**b**) pure nitrate spectra for the same region at the same concentrations; (**c**) secondary peak of nitrite spectra; (**d**) secondary peak of nitrate spectra; and (**e**) pure NO_x_^−^ spectra for different concentrations of nitrate and nitrite, used in developing the SVR model.

The estimated NO_x_^−^ spectra presented in Figure 7 were passed to the machine learning model developed using the SVR algorithm to predict the relative NO_x_^−^ concentrations. For this purpose, nitrate and nitrite solutions of known concentrations were mixed to form several combinations, and the corresponding NO_x_^−^ spectra were acquired and used in model training. Figure 8a–e shows the individual nitrite, nitrate, and mixed NO_x_^−^ spectra at various concentrations in Milli-Q water, where it is evident that for the same concentration, nitrate has a relatively higher absorbance than nitrite. The peak absorption of nitrite spectra appears at 210 nm, which is approximately symmetric with respect to concentration (Figure 8a). On the other hand, the peak absorption for nitrate spectra starts from 205 nm and gradually shifts to the right as the concentration increases (Figure 8b). For a relatively high concentration of nitrite and nitrate (over 10 mg-N L^−1^), a secondary peak was visible at 302 nm and 355 nm, respectively (Figure 8c,d). However, for low concentrations (below 10 mg-N L^−1^), the UV absorption at the secondary peak region is inadequate, hence, they cannot be effectively applied to quantify the low level of nitrite and nitrate usually encountered in drinking water during nitrification. Spectral analysis suggests that the shape of the NO_x_^−^ spectra is primarily dominated by the nitrate concentration, as it has a relatively higher absorbance compared to nitrite (Figure 8e). Using the NO_x_^−^ spectra as predictor variables, and corresponding concentrations as response variables, the SVR model requires a fine tune of the two parameters. The parameter C represents the penalty for misclassified data points, whereas the parameter Υ controls the curvature of the decision boundary. They were obtained by the grid search method, and the RBF kernel was used to map the data. Using a 10-fold cross-validation, the performance of the SVR model, in terms of R^2^ in model training and cross-validation, was over 0.99, with RMSE < 0.04, which indicates a satisfactory relationship between spectra and concentration. The final parameters of the SVR model are presented in Table A2 in Appendix A. The developed SVR model was used to predict the NO_x_^−^ concentrations in the six samples using the separated NO_x_^−^ spectra presented in Figure 7.

**Figure 9:**
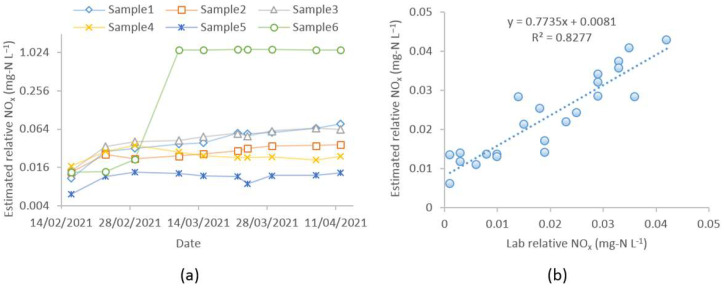
(**a**) Estimated relative NO_x_^−^ concentration (mg-N L^−1^) by the proposed method; (**b**) linear relationship between the estimated and the lab data (the data point in Sample6 after nitrification occurrence was not included in the plot, considering the fact that it has a relatively higher NO_x_^−^ value, which can affect the trend line).

**Figure A1:**
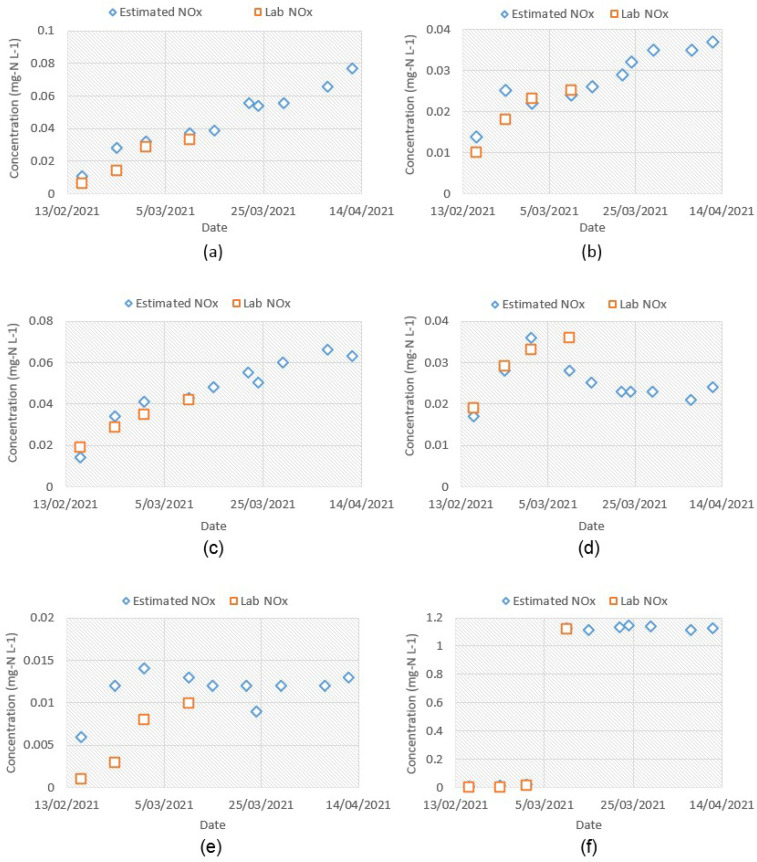
Plot of predicted and lab relative NOx concentrations: (**a**) Sample1; (**b**) Sample2; (**c**) Sample3; (**d**) Sample4; (**e**) Sample5; (**f**) Sample6.

The estimated NO_x_^−^ concentration by the proposed method is shown in Figure 9a, and the numeric values are presented in Table A3 in Appendix A. The NO_x_^−^ values in the figure represent the relative NO_x_^−^ concentration with respect to the baseline values, as the initial NO_x_^−^ content in the baseline spectra was set to zero. Therefore, the total NO_x_^−^ concentration would be the sum of initial NO_x_^−^ content in the baseline spectra plus relative NO_x_^−^ content estimated by the method. In Figure 9a, Samples 1–3 show an increasing trend in NO_x_^−^ production, whereas Samples 4–5 show an initial increase, followed by either a stable or decreasing trend. On the other hand, the trend of Sample6 shows a sharp rise in NO_x_^−^ concentration, indicating a nitrification episode. Overall, the estimated NO_x_^−^ concentrations for all samples closely reflect their spectra presented in Figure 7. The proposed method was validated against the lab NO_x_^−^ data (Appendix A, Table A4 and Table A5), and a good level of agreement was found between them. Figure A1 in Appendix A compares the estimated relative NO_x_^−^ concentration by the proposed method and the relative NO_x_^−^ concentrations calculated from the lab data. Figure 9b shows a strong linear relationship (R^2^ = 0.83) between the estimated and the lab data. The trend-line equation represents the local calibration function to relate the lab data with the model predicted values. Some inaccuracies may be encountered during the estimation of the ∆_i, sample_ and corresponding NO_x_^−^ spectra by the linear models. Also, if the time difference between the spectra fingerprint and the lab test result varies, local calibration performance can be poor.

The literature and the guidance manuals of many water utilities suggest that changes in nitrite by 0.05 mg-N L^−1^ or above gives an indication of nitrification [34]. The proposed method tracks the change of relative NO_x_^−^ concentration, hence, a value around 0.05 mg-N L^−1^ indicates either an increased level of nitrite or nitrate or both. In such a case, further investigation is required to ensure the status of nitrification.

## 4. Discussion

The spectrophotometric method proposed in this paper can be implemented by the drinking water industry to monitor nitrification activity throughout the network. Implementing this method online will require regular changing of the baseline to cope with the incoming water. In a drinking water distribution system, the quality of water and spectral characteristics change continuously, depending on source and treatment characteristics. To obtain more accurate estimates of NO_x_^−^, a local calibration using site-specific water quality data is recommended. The accuracy of the laboratory measurements may also be subject to errors from various sources, including measurement range, analysis method, human errors in grab sampling, etc. This is a critical part, as the accuracy of the method is highly affected by the level of calibration. Therefore, calibration using good water quality data is required to obtain a better accuracy by the proposed method. 

The lab UV-Vis fingerprint was obtained by filtering the sample through 0.45 μm filter paper to remove the particle absorbances. However, in recent years, several chemometric techniques have been developed to do the particle compensation [14,35]. It is recommended to compare both methods to improve the particle compensation. The chemometric particle compensation techniques could be possibly incorporated into the method proposed in this paper to develop an automated nitrification monitoring system. This could be an area to investigate further. 

**Figure 10:**
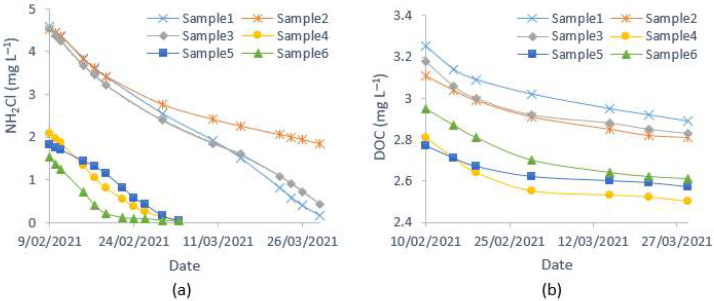
Observed water quality variations during the study period: (**a**) monochloramine and (**b**) DOC.

It was assumed that the absorbance reduction at 245 nm was entirely caused by monochloramine decay. However, at that wavelength, NOM may contribute towards absorbance reduction. As shown in Figure 10, monochloramine decay during the study period was much faster than the decrease of organic concentration (measured as DOC), which was ≤ 0.2 mg L^−1^. When this method is employed within a short period (usually less than a week), spectra change due to organic absorbance at 245 nm can be considered to be zero. Therefore, this method works well within a short period. Otherwise, if the DOC concentration is available, it can be used to quantify the absorbance reduction by NOM, since DOC is reported to have an excellent correlation to UV absorbances. Moreover, due to monochloramine autodecomposition, free ammonia is produced, which may also absorb UV light. The spectral analysis of the pure ammonia solution indicated that it has little or no UV absorbances when appearing in a relatively low concentration, usually found in drinking water systems (up to 5 mg L^−1^ as NH_3_-N).

The spectral fingerprints were taken using a 10 cm optical pathlength cell, and the maximum NO_x_^−^ concentration used to build the SVR model was 2.4 mg-N L^−1^ (1.2 mg-N L^−1^ of nitrite + 1.2 mg-N L^−1^ of nitrate), hence, the method works well within that limit. Beyond that limit, the instrument sensitivity may affect the measurement accuracy. If a high concentration of nitrate and nitrite are expected, it is recommended to use a short pathlength cell. Also, it was assumed that the major light-absorbing species in the spectra were NOM, nitrate, nitrite, and monochloramine, which are common in most typical drinking waters. No chemical reaction between these species, or absorbance increase/decrease due to their internal reactions, were considered. For a relatively short time duration between the baseline and the subsequent spectra, the internal reactions and their effects on the spectra can be negligible. If other UV light-absorbing species exist in the spectra, the accuracy of the method may differ due to the interferences by these species. Further study is required to confirm this.

Currently, UV spectrophotometry has been increasingly used for online monitoring of various water quality parameters [1]. Among the various methods of nitrification monitoring, spectroscopic methods are preferable because of their simplicity, rapid detection, and high sensitivity, which improves the accuracy of detection [2,36]. To develop online based sensors, Banna et al. [37] indicated important criteria, including response, measurement range, sensitivity, accuracy, lifetime, maintenance, and cost, need to be considered. The proposed method has a rapid response with high sensitivity and good accuracy, hence, it can be used to develop an online nitrification monitoring system. Unlike many other UV-based methods of nitrate/nitrite monitoring of environmental samples which use oxidizing or complexing agents, this method is simple and does not require any chemical addition. Compared to other methods, such as SDA, the incorporated spectral compensation for monochloramine absorbance makes it more compatible for monitoring rapid chloramine decay and nitrification in chloraminated systems. By incorporating the online monitoring to the proposed method, an early warning system can be developed that will help to prevent nitrification in drinking water distribution systems. This could be an area to explore further.

## 5. Conclusions

A novel method of monitoring nitrification in drinking water is proposed in this paper. In typical chloraminated drinking water, major UV light-absorbing substances are NOM, nitrate, nitrite, and monochloramine. Both nitrate and nitrite absorb UV light in the same wavelength range, hence, nitrification activity can be monitored by assessing the combined NO_x_^−^ spectra. When complete or incomplete nitrification occurs, the spectra fingerprint is changed due to the change of concentrations of these species. With some simple assumptions, the NO_x_^−^ spectra were isolated from the total spectra to estimate its NO_x_^−^ content. The spectral analysis for a number of test samples over several weeks suggest that maximum downward spectral shift was due to monochloramine decay at 245 nm wavelength. In contrast, the spectra shift slightly upward between 200 nm and 225 nm wavelengths due to the production of NO_x_^−^. Since both monochloramine and NO_x_^−^ have an effect on spectra change in between 200 nm and 245 nm, pure monochloramine spectra were investigated to quantify its individual effect. The analysis of pure monochloramine spectra at various concentrations indicated that the absorbance difference between two concentrations at 245 nm ($\Delta_{245}$) is related to other wavelengths ($\Delta_{i}$) by a linear function. Using these linear models established in ultrapure water, the $\Delta_{i}$ values for the test sample were calculated based on its $\Delta_{245}$ value. 

To work with this method, the test sample is required to keep for a certain period to allow monochloramine decay and NO_x_^−^ production. The spectra fingerprint is recorded at the beginning (t = 0), considered as baseline, and at the end (t = t). The estimated $\Delta_{i}$ values are subtracted from the baseline to approximate the spectra change caused by monochloramine decay only. Finally, subtracting these estimated spectra from the spectra at time t will give the NO_x_^−^ spectra for that time duration.

A dataset was created using a range of NO_x_^−^ spectra and corresponding concentrations in ultrapure water. The dataset was used to train a machine learning model using the support vector regression (SVR) algorithm. The detection accuracy by the model was tested for different kernel functions, and the radial basis function was found to have excellent accuracy. The SVR model was used to predict the relative NO_x_^−^ concentrations of the test samples. Finally, the following points can be summarized:

- Nitrification in chloraminated water can be monitored by isolating and modelling the NO_x_^−^ spectra
- The level of precision by the proposed method is ± 0.01 mg-N L^−1^

## Figures and Tables

**Table 1:** The average value (mean ± 1 SD) of water quality parameters at the TB-WTP and the studied points in the distribution system.

Water Quality Variables	TB-WTP	Meningie
pH	8.5 ± 0.2	8.5 ± 0.3
Turbidity (NTU)	0.10 ± 0.01	0.15 ± 0.10
Color (HU)	1 to 2	1 to 2
DOC (mg L^−1^)	2.4 ± 0.26	2.50 ± 0.90
Free chlorine (mg L^−1^)	<0.10	<0.10
Monochloramine (mg L^−1^)	4.10 ± 0.30	1.60 ± 1.0
Dichloramine (mg L^−1^)	<0.10	<0.10
Free ammonia (NH_3_-N) (mg L^−1^)	0.25 ± 0.09	0.40 ± 0.06
Nitrate + Nitrite (NO_x_^−^-N) (mg L^−1^)	0.10 ± 0.04	0.30 ± 0.13

## Appendix A

**Table A1:** R-Square and RMSE values in linear model fit between ∆_245, ultrapure_ and ∆_i, ultrapure_.

Wavelength	R-Sq.	RMSE	Wavelength	R-Sq.	RMSE	Wavelength	R-Sq.	RMSE
200	0.96	0.011	215	0.971	0.004	230	1	0.001
200.5	0.958	0.011	215.5	0.974	0.004	230.5	1	0.001
201	0.957	0.01	216	0.977	0.004	231	1	0.001
201.5	0.955	0.009	216.5	0.979	0.004	231.5	1	0.001
202	0.954	0.009	217	0.982	0.004	232	1	0.001
202.5	0.952	0.008	217.5	0.983	0.004	232.5	1	0.001
203	0.95	0.008	218	0.985	0.003	233	1	0.001
203.5	0.948	0.008	218.5	0.987	0.003	233.5	1	0.001
204	0.946	0.007	219	0.989	0.003	234	1	0.001
204.5	0.944	0.007	219.5	0.99	0.003	234.5	1	0.001
205	0.942	0.007	220	0.991	0.003	235	1	0.001
205.5	0.94	0.007	220.5	0.992	0.003	235.5	1	0
206	0.938	0.007	221	0.993	0.003	236	1	0
206.5	0.937	0.007	221.5	0.994	0.003	236.5	1	0
207	0.936	0.006	222	0.995	0.003	237	1	0
207.5	0.936	0.006	222.5	0.996	0.002	237.5	1	0
208	0.937	0.006	223	0.996	0.002	238	1	0
208.5	0.938	0.006	223.5	0.997	0.002	238.5	1	0
209	0.939	0.006	224	0.997	0.002	239	1	0
209.5	0.94	0.006	224.5	0.998	0.002	239.5	1	0
210	0.942	0.005	225	0.998	0.002	240	1	0
210.5	0.944	0.005	225.5	0.998	0.002	240.5	1	0
211	0.947	0.005	226	0.999	0.002	241	1	0
211.5	0.95	0.005	226.5	0.999	0.002	241.5	1	0
212	0.953	0.005	227	0.999	0.002	242	1	0
212.5	0.956	0.005	227.5	0.999	0.002	242.5	1	0
213	0.959	0.005	228	0.999	0.001	243	1	0
213.5	0.963	0.005	228.5	0.999	0.001	243.5	1	0
214	0.966	0.004	229	0.999	0.001	244	1	0
214.5	0.969	0.004	229.5	1	0.001	244.5	1	0

**Table A2:** SVR model details.

SVR Parameters	
SVM-Type	Eps-regression
SVM-Kernel	Radial basis function
Cost	1040
Gamma	0.01098901
Epsilon	0.001
Number of Support Vectors	176

**Table A3:** Predicted relative NOx concentration using the SVR model.

Date	Sample1	Sample2	Sample3	Sample4	Sample5	Sample6
9/02/2021	0	0	0	0	0	0
16/02/2021	0.011	0.014	0.014	0.017	0.006	0.013
23/02/2021	0.028	0.025	0.034	0.028	0.012	0.014
1/03/2021	0.032	0.022	0.041	0.036	0.014	0.021
10/03/2021	0.037	0.024	0.043	0.028	0.013	1.126
15/03/2021	0.039	0.026	0.048	0.025	0.012	1.113
22/03/2021	0.056	0.029	0.055	0.023	0.012	1.134
24/03/2021	0.054	0.032	0.050	0.023	0.009	1.144
29/03/2021	0.056	0.035	0.060	0.023	0.012	1.139
7/04/2021	0.066	0.035	0.066	0.021	0.012	1.119
12/04/2021	0.077	0.037	0.063	0.024	0.013	1.127

**Table A4:** Lab NOx data (mg-N L^−1^).

Date	Sample1	Sample2	Sample3	Sample4	Sample5	Sample6
9/02/2021	0.134	0.131	0.14	0.145	0.183	0.24
16/02/2021	0.14	0.141	0.159	0.164	0.184	0.241
23/02/2021	0.148	0.149	0.169	0.174	0.186	0.243
1/03/2021	0.163	0.154	0.175	0.178	0.191	0.255
10/03/2021	0.167	0.156	0.182	0.181	0.193	1.36

**Table A5:** Relative NOx production between the first row and the subsequent rows in Table A4 (mg-N L^−1^).

Date	Sample1	Sample2	Sample3	Sample4	Sample5	Sample6
9/02/2021	0	0	0	0	0	0
16/02/2021	0.006	0.01	0.019	0.019	0.001	0.001
23/02/2021	0.014	0.018	0.029	0.029	0.003	0.003
1/03/2021	0.029	0.023	0.035	0.033	0.008	0.015
10/03/2021	0.033	0.025	0.042	0.036	0.01	1.12
